# Advancing pain management for extremity trauma: the evolution of ultrasound-guided nerve blocks for patients in the supine position in trauma centers

**DOI:** 10.1007/s00068-024-02523-w

**Published:** 2024-04-22

**Authors:** Yuefeng Huaguo, Shuai Kang, Li Hu, Hongmei Zhou

**Affiliations:** 1 Department of Anesthesiology, The Second Affiliated Hospital of Jiaxing University, Huancheng Strasse 1518, Jiaxing City, 314000 China; 2 Key Laboratory of Basic Research and Clinical Transformation of Perioperative Precision Anesthesia, Jiaxing City, China

**Keywords:** Nerve block, Trauma centers, Pain management

## Abstract

**Purpose:**

Trauma, particularly extremity trauma, poses a considerable challenge in healthcare, especially among young adults. Given the severity of patient pain and the risks associated with excessive opioid use, managing acute pain in trauma centers is inherently complex. This study aims to investigate the application and benefits of ultrasound-guided nerve blocks for early pain management in patients with extremity trauma positioned supine.

**Methods:**

A comprehensive literature review was conducted to assess the effectiveness and advantages of ultrasound-guided peripheral nerve blocks in the acute pain management of extremity trauma patients in the supine position. Special emphasis was placed on evaluating the selection criteria, indications, contraindications, adverse reactions, and potential complications associated with these nerve block techniques.

**Results:**

Ultrasound-guided nerve blocks represent a safer and more precise option for managing pain in extremity trauma patients placed in the supine position. These techniques offer significant advantages in terms of reducing healthcare expenses, diminishing reliance on opioid medications, and mitigating opioid-related complications. Nonetheless, challenges may arise due to the necessity for patient cooperation during specific nerve block procedures.

**Conclusion:**

Ultrasound-guided nerve blocks present a promising avenue for early pain management in extremity trauma patients positioned supinely. Their implementation can lead to improved patient outcomes by alleviating pain severity, reducing opioid consumption, and cutting down healthcare costs. Further research and clinical integration of these techniques is imperative to enhance pain management protocols in trauma centers.

## Introduction

According to literature statistics from the World Health Organization (WHO), 10% of deaths and 16% of disabilities worldwide are caused by trauma [1]. More critically, it has become the leading cause of death in individuals under the age of 44 [2]. High-energy injury factors such as road traffic accidents and falls from heights have led to a significant increase in the occurrence of polytrauma and critically injured trauma patients, most of whom require emergency debridement or orthopedic surgery [3]. A survey from China showed that the extremities were the most common site of injury, representing approximately 88.05% of all injury sites [4]. Besides the high mortality rate associated with acute trauma, pain management following trauma poses a substantial challenge.

Trauma patients experience intense stress responses due to severe pain during various stages such as examination, investigation, transportation, and preoperative preparation. This results in the abnormal release of a significant number of inflammatory mediators, causing severe discomfort in patients and possibly leading to serious complications [5]. Untreated pain has been shown to increase the incidence of diseases such as chronic pain and post-traumatic stress disorder [6]. For conditions like extremity fractures that cause severe pain, traditional intravenous and oral analgesics often fail to provide effective pain relief. Moreover, systemic analgesics can potentially mask pain from other injured areas, leading to delayed diagnosis and treatment [7].

A multitude of nerve block procedures have been used to provide analgesia and surgical anesthesia. However, some nerve blocks require the patient’s cooperation to change the position, which may result in severe pain. In this context, performing nerve blocks in extremity trauma patients in the supine position by anesthesiologists may represent a safer and more effective approach to pain management. This review discusses the advantages, indications, contraindications, choice of nerve blocks for different sites of extremity trauma, and associated complications of ultrasound-guided anterior supine nerve blocks in the context of trauma center multidisciplinary collaboration.

## Advantages of nerve blocks

### Pain reduction

Peripheral nerve blocks involve the local administration of anesthetic agents around nerves, temporarily interrupting sensory transmission while preserving motor function to varying degrees. In recent years, with the continuous development of ultrasound technology, peripheral nerve block anesthesia can be visualized under ultrasound guidance [8]. Better analgesia of the extremity injury may allow for improved monitoring and serial exams of patients with other areas of concern. They reduce pain stimuli, improve the patient’s psychological state, alleviate psychological stress reactions, and relieve tension, anxiety, and other negative emotions caused by pain [9–11]. Alkassabi et al. conducted a systematic review of risk factors for persistent pain after musculoskeletal trauma and found that early high-intensity acute pain is a significant factor in the development of chronic pain [12]. Given the effectiveness of peripheral nerve blocks in reducing acute pain, they may play a role in reducing the severity of early pain, which, in turn, affects the development of chronic pain.

### Reduced opioid use

Opioid medications are essential for acute pain management, but they come with adverse effects such as respiratory depression, hypotension, nausea, delirium, and vomiting [13]. Long-term use also increases the risk of drug dependence and addiction [14], placing limitations on their usage. With the widespread adoption of Enhanced Recovery After Surgery (ERAS) principles, multimodal analgesia has become a crucial component of perioperative pain management [15]. Peripheral nerve blocks are an integral part of multimodal analgesia. A prospective randomized controlled trial has shown significant advantages of implementing iliac fascia blocks for pain management in elderly patients with hip fractures in terms of pain relief, reducing opioid-related side effects, and shortening hospital stays [16]. Another advantage of peripheral nerve blocks is that they serve as an effective alternative for pain control in patients with chronic pain, opioid tolerance, or contraindications to opioid use.

### Reduced occurrence of delirium

Delirium is an acute cognitive disorder characterized by acute and fluctuating impairments in attention and consciousness [17]. Research has indicated that delirium may lead to long-term declines in patients’ cognitive, physiological, and social functioning, increase medical costs, and raise mortality rates and the incidence of postoperative complications [18]. The use of opioid medications has been associated with delirium in elderly orthopedic trauma patients. The mechanisms through which peripheral nerve blocks reduce delirium occurrence may be diverse and include pain improvement, decreased opioid use, enhanced analgesic effects, and more [19]. Studies have demonstrated that pre-hospital peripheral nerve blocks in elderly hip fracture patients have been shown to effectively reduce pain and delirium occurrence compared to traditional pain management methods [20, 21].

### Shortened hospital stays and reduced hospital costs

Severe pain resulting from orthopedic trauma often requires a significant amount of medication for relief, which may increase the risk of unexpected opioid-related side effects and potentially prolong patient hospital stays. Peripheral nerve blocks are one of the options that healthcare institutions seek to reduce these complications. In a randomized controlled trial, preoperative pain management with iliac fascia blocks in ultra-elderly patients with hip fractures demonstrated a significant reduction in the average length of hospital stay for patients receiving iliac fascia blocks [22]. Another prospective study comparing procedural sedation and ultrasound-guided brachial plexus blocks for pain control in patients with acute shoulder dislocation indicated a shorter emergency department stay for patients receiving peripheral nerve blocks [23]. In patients with shoulder dislocation, avoiding procedural sedation eliminated the need for prolonged monitoring of vital signs in the emergency department. Stone et al.’s study comparing peripheral nerve blocks and procedural sedation in patients with upper limb fractures, abscesses, and joint dislocations showed that patients in the peripheral nerve block group had an average emergency department stay of 106 min, while the intravenous sedation group had a stay of 285 min [23]. Prolonged hospitalization also leads to increased medical costs. Procedural sedation requires specialized medical personnel for operation and stricter monitoring, leading to increased complications associated with procedural sedation, such as reflux aspiration, respiratory depression, and hypotension, all of which can subsequently increase healthcare-related costs [24].

## Indications and contraindications of ultrasound-guided nerve blocks for patients with acute extremity trauma

Upon arrival of trauma patients at the hospital trauma center, comprehensive assessments are performed by emergency physicians. Patients with Visual Analogue Scale (VAS) scores of ≥ 6, as evaluated by trauma center emergency physicians, are considered for ultrasound-guided nerve blocks after excluding contraindications and obtaining informed consent from patients or their guardians.

The absolute contraindications to performing a peripheral nerve block include the following conditions: (1) the patient refuses; (2) patients with severe mental disorders or inability to cooperate with the operation, as this may lead to improper or unsafe operation; (3) history of severe allergic reaction to the local anesthetic or other injectable drugs used, as this may lead to the occurrence of allergic reactions, including severe anaphylactic shock; (4) patients with multiple injuries and unstable vital signs; for example, patients who have combined with rupture and bleeding of abdominal parenchymal organs; (5) presence of severe cardiovascular disease, such as severe arrhythmias and cardiac insufficiency, as this may lead to the development of cardiovascular events, such as cardiac arrest [25].

The relative contraindications include the following conditions: (1) pregnant women, because of their altered physiological state, which puts them at increased risk of toxic reactions to local anesthetics and makes resuscitation more difficult, need to choose their local anesthetic drugs and doses carefully, and early recognition and prompt treatment are key to avoiding serious complications caused by local anesthetic toxicity [26]; (2) patients with coagulopathy or who are on anticoagulant therapy, because this may increase the risk of bleeding, especially when performing a deep nerve block when bleeding can lead to catastrophic results, and superficial peripheral nerve blocks can be considered in the presence of mild coagulopathy after appropriate discussion of the risks and benefits with the patient or surrogate [27, 28]; (3) patients with local anatomical abnormalities or tumors affecting the block area, as this may lead to a poor block or damage to surrounding structures; (4) patients with severe respiratory disease or respiratory failure, which is a key factor in such patients receiving a interosseous groove brachial plexus nerve block may result in respiratory insufficiency; (5) infection close to the puncture site, as this may increase the risk of systemic infection [29]; (6) patients with neurologic disease, either subclinical or overt pre-existing neuropathy may render these patients susceptible to long-term nerve damage [30, 31].

## Selection of ultrasound-guided nerve blocks

### Upper limb injuries

Upper limb fractures or dislocations are common in trauma centers, and ultrasound-guided brachial plexus nerve blocks are frequently used in the perioperative anesthesia management of upper limb fractures. Various approaches can be chosen. Interscalene brachial plexus nerve blocks are commonly used in clinical practice and widely applied for anesthesia and postoperative analgesia for upper limb and shoulder surgeries. However, they have the disadvantage of incomplete ulnar nerve block and ipsilateral phrenic nerve block. For some trauma patients who are obese or have concomitant chronic obstructive pulmonary disease, phrenic nerve block causes diaphragmatic paralysis and can lead to chest discomfort or exacerbating symptoms in patients with other pulmonary comorbidities [32]. Performing interscalene brachial plexus nerve blocks in the emergency department for such patients may lead to adverse events. Therefore, it may be necessary to choose other accesses to perform brachial plexus blocks in these patients.

The supraclavicular brachial plexus nerve block results in anesthesia of the upper limb that includes the shoulder [33]. It is known as the “spinal of the arm.” Compared with interscalene block, it can complete ulnar nerve block, and much lower proportion of phrenic nerve blocks [34]. Research by Stone et al. indicates that ultrasound-guided supraclavicular brachial plexus nerve blocks performed in the emergency department shorten the length of stay and do not affect the safety and satisfaction of patients undergoing upper limb fracture, dislocation, or abscess surgery compared to local infiltration [24]. But it also has some disadvantage, include pneumothorax, and recurrent laryngeal nerve blockade leading to hoarseness [35, 36].

The axillary block can anesthetize the median nerve, the ulnar nerve, the radial nerve, and the musculocutaneous nerve, resulting in anesthesia of the upper limb from mid-arm extending distally to the elbow, forearm, and hand. In order to perform this block, the patient is positioned supine with the arm abducted to 90°. It may be difficult for patients with upper extremity injuries to abduct the arm. And the axillary nerve block carries the risk of hematoma formation and intravascular injection, because of its close proximity to the axillary artery and vein. The need to abduct the arm to perform the axillary block may be difficult with certain upper extremity injuries [37].

Costoclavicular brachial plexus nerve blocks are a new type of brachial plexus nerve block in recent years. Research shows that this approach is easily distinguishable under ultrasound, with a low variability in vascular and nerve positions. It requires about 8–26 mL dose of local anesthetic (e.g., 0.5% ropivacaine) for satisfactory blocking [38]. The incidence of diaphragmatic paralysis caused by phrenic nerve block is significantly lower than that of supraclavicular brachial plexus nerve blocks. It also has the advantages of rapid onset and high success rate [39–42].

Shoulder dislocation is one of the most common joint dislocations in clinical practice, often occurring in elderly individuals and accompanied by severe pain and joint deformity. Treatment involves pain relief and early reduction [43]. Anesthesia options for patients with shoulder dislocation who undergo manual reduction surgery can include brachial plexus nerve blocks or general anesthesia. General anesthesia requires sufficient fasting time for patients. A case series research shows that selective suprascapular nerve blocks can provide satisfactory postoperative analgesia for patients undergoing shoulder surgery and can effectively reduce the occurrence of phrenic nerve block–induced diaphragmatic paralysis [44]. Therefore, selective suprascapular nerve blocks may be an ideal choice for early pain relief or manual reduction in patients with shoulder dislocation.

The intercostobrachial nerve (ICBN) arises from the collateral branch of the second intercostal nerve [45]. It mainly dominates the skin of the medial side of the arm, the axillary, and the lateral chest wall. ICBN block combined with brachial plexus block can relieve pain caused by upper limb surgical and tourniquet [46, 47].

For certain upper limb distal injuries, ultrasound-guided single-injection brachial plexus nerve blocks may be a preferable option. Isfahani demonstrated that radial nerve blocks, such as the posterior interosseous nerve block, provide excellent analgesia for distal radius fractures in the emergency department [48]. There is evidence that ultrasound-guided forearm nerve blocks have been shown to safely reduce pain for emergency procedures in the emergency department [49]. One randomized controlled trial indicated ultrasound-guided ulnar, median, and radial nerve blocks are effective for pediatric patients in the ED [50]. The procedure provides effective analgesia and facilitates care while minimizing iatrogenic risk.

### Lower limb injuries

Lower limb fractures being the most common injuries in pre-hospital trauma patients caused by traffic accidents [51]. These patients often experience severe pain, especially when changing positions. Additionally, they may require emergency wound debridement or internal and external fixation surgeries. There are various options for peripheral nerve blocks for lower limb injuries.

The lumbar plexus is an important nerve plexus that supplies the hip, femur, and knee joints, comprising the anterior branches of the T12 to L4 spinal nerves. Its branches include the obturator nerve, lateral femoral cutaneous nerve, femoral nerve, ilioinguinal nerve, iliohypogastric nerve, and genitofemoral nerve [52]. Lumbar plexus nerve block is essential for lower limb fracture surgery and perioperative pain management. However, posterior lumbar plexus block may lead to various complications, such as spinal or epidural anesthesia, nerve damage, local anesthetic toxicity, and retroperitoneal hematoma [37]. Patients also need to cooperate by assuming specific positions, which can be painful. Supine-position lumbar plexus nerve block, proposed by Liu et al. in 2018, is a new approach [53]. A randomized controlled trial that included a total of 126 patients showed that supine-position lumbar plexus nerve block can achieve good analgesic effects. Compared to lateral lumbar plexus nerve block, patients undergoing supine-position lumbar plexus nerve block have higher satisfaction, better nerve identification and localization, shorter procedure times, and a lower risk of epidural blockade, making it a safer and more convenient approach [54].

Iliacus fascia block is also a commonly used method. This approach can block important branches of the lumbar plexus, such as the femoral nerve, lateral femoral cutaneous nerve, obturator nerve, and genitofemoral nerve. Hip joint fractures are more common in elderly trauma patients and significantly impact their quality of life. Although fractures are usually moderately to severely painful, patients with hip joint fractures are often reluctant to use analgesic drugs preoperatively due to concerns about delirium, respiratory depression, and potential undiagnosed fractures [55]. Recent studies have shown that early ultrasound-guided femoral nerve block or iliacus fascia block can significantly reduce pain scores, reduce the need for analgesic drugs, and facilitate patient positioning [56]. According to a study, pre-hospital iliac fascia plane block can reduce patients’ perioperative resting and movement pain, inflammatory responses, and delirium, as well as shorten hospital stays [57]. Numerous studies have demonstrated that iliac fascia plane block is a safe, fast, and easy blockade method [58, 59].

The femoral nerve is one of the major branches of the lumbar plexus, providing analgesia for injuries to the anterior inner thigh, femur, knee, lower leg, and inner ankle. In cases of extensive lower limb injuries, tourniquets may be applied in the emergency department to reduce bleeding. However, prolonged tourniquet use can cause pain and restlessness in patients, as well as significant hemodynamic fluctuations [60]. Studies have shown that ultrasound-guided iliac fascia plane block or femoral nerve block can effectively alleviate tourniquet-related discomfort [61, 62]. This not only reduces patient suffering but also effectively reduces cardiovascular adverse events.

The saphenous nerve is the largest branch of the femoral nerve, providing sensory innervation to the medial knee and the inner side of the lower leg and foot. It is often used in combination with sciatic nerve block for injuries or surgeries of the foot and ankle, providing complete motor and sensory nerve supply to the ankle. In a randomized controlled trial conducted by Sort, involving a cohort of 150 patients, findings demonstrated that the utilization of ultrasound-guided popliteal sciatic nerve block in conjunction with saphenous nerve block, as opposed to intrathecal anesthesia, proved to be efficacious in providing postoperative analgesia for individuals undergoing internal fixation for ankle fractures. This approach was associated with a diminished requirement for postoperative opioids, ensuring a high level of safety, and eliciting a notable enhancement in patient satisfaction by 45% [63].

The sciatic nerve originates from the sacral plexus, dividing into the tibial nerve and common peroneal nerve at the popliteal fossa, supplying sensation and motor function to the lower leg and dorsal foot except for the inner side. Sciatic nerve blocks are widely used for anesthesia and perioperative pain management in lower limb surgery, with common approaches being anterior and posterior. The posterior approach often requires patients to assume a lateral decubitus position, which can be painful. For lower limb trauma patients, the anterior approach to sciatic nerve block may be a better choice. However, traditional anterior sciatic nerve block has limitations, and studies have shown that it is a technically challenging procedure with high operator requirements and a high failure rate [64, 65]. Zou Yinghua et al. validated an improved anterior sciatic nerve block approach in which the puncture needle is nearly perpendicular to the ultrasound beam during the puncture process, making the needle visualization clearer, enhancing the safety of the puncture process, and providing nerve block effects comparable to traditional anterior sciatic nerve block [62]. Zhu et al., through the lateral approach at the upper outer thigh, demonstrated that this approach has a higher puncture success rate, better analgesic effects, and similar safety to the traditional approach [66].

## Adverse reactions and complications of ultrasound-guided nerve blocks in trauma centers

### Incomplete blocks

Compared to upper limb nerve blocks, lower limb nerve blocks are deeper and involve larger nerves, making them more challenging and prone to incomplete blocks [67]. Research by He Zhengguang et al. suggests that a high BMI is an independent risk factor for incomplete lower limb nerve blocks [68]. Additionally, a study from Japan has shown that obesity is a cause of incomplete blocks [69]. Patients with higher BMI values have thicker adipose tissue, deeper nerve locations, and increased difficulty in visualizing the needle tip in adipose tissue during ultrasound-guided nerve blocks, making ultrasound localization more difficult and increasing the risk of incomplete blocks.

### Masking patient conditions

One of the primary concerns in administering early analgesic treatment to trauma patients is the potential for pain relief to obscure the patient’s condition, leading to delayed diagnosis and severe consequences. Extremity compartment syndrome is a severe complication in trauma patients, especially those with tibia fractures [70]. The diagnosis of compartment syndrome primarily relies on clinical symptoms and signs. However, according to relevant reports, clinical signs have low sensitivity and positive predictive value but high specificity and negative predictive value [7]. Existing literature on compartment syndrome lacks prospective, randomized controlled study results and lacks information-rich meta-analyses. Therefore, for trauma patients, rather than debating whether to perform peripheral nerve blocks for limb pain management, it is more important to focus on closely monitoring the use of analgesics, the occurrence of breakthrough pain, and monitoring compartment pressures in high-risk patients [71]. Vigilance on the part of surgical, anesthesia, and nursing teams involved in trauma patient care at all trauma centers is crucial for the early detection of compartment syndrome [72].

Relevant guidelines also emphasize that pain relief is a fundamental right of patients [73]. As long as patients are closely monitored, the use of low-concentration local anesthetics for single-shot peripheral nerve blocks under ultrasound guidance for limb pain management does not delay the diagnosis of the patient's condition.

### Local anesthetic toxicity reactions

Although ultrasound-guided nerve blocks have become a widely used and safe technique in clinical practice, local anesthetic toxicity reactions remain a common adverse event that requires attention. The incidence of overall local anesthetic toxicity reactions is approximately 2–2.8 per 10,000 cases [74]. While the incidence is relatively low, severe local anesthetic toxicity reactions can be life-threatening. The occurrence of local anesthetic toxicity reactions in patients is related to the presence of risk factors and the type of local anesthetic used. Factors contributing to local anesthetic toxicity reactions include direct injection into blood vessels, overdosing, or injection into highly vascularized areas, leading to increased absorption of local anesthetics over a short period [75]. The diagnosis of local anesthetic toxicity reactions relies primarily on clinical manifestations following drug administration. Therefore, close patient monitoring is essential. The major clinical manifestations of local anesthetic toxicity reactions involve the central nervous system and the cardiovascular system. However, before the onset of typical seizures and circulatory arrest, patients often experience prodromal symptoms, such as perioral numbness and metallic taste. Hence, vigilant monitoring and early recognition of these prodromal symptoms are essential to prevent serious consequences associated with local anesthetic toxicity reactions [71]. In the context of trauma centers providing early analgesic treatment to trauma patients using ultrasound-guided peripheral nerve blocks, it is crucial to acknowledge that some centers may lack the necessary monitoring equipment and adequate healthcare staff for close patient surveillance. This could potentially lead to the oversight of early clinical signs related to local anesthetic toxicity reactions and result in severe consequences. Therefore, knowledgeable and skilled physicians should perform nerve blocks in trauma centers and select local anesthetics with a higher safety profile while ensuring effective monitoring.

### Rebound pain after peripheral nerve blocks

In recent years, researchers have proposed the concept of “rebound pain” following peripheral nerve blocks, which is a relatively common yet not fully understood acute pain that occurs within 24 h after the resolution of the effects of nerve blocks [76]. Its primary clinical manifestations include sudden severe pain, often described as stabbing, electric shock–like, or numbness, with a Numerical Rating Scale (NRS) score of ≥ 7 [77]. Risk factors for the occurrence of rebound pain after peripheral nerve blocks may include young age, female gender, orthopedic surgery, and perioperative non-use of dexamethasone [76]. Despite the occurrence of rebound pain after nerve blocks, patients generally express high satisfaction with the nerve blocks and remain willing to undergo them [78]. Therefore, in clinical practice, measures should be taken to reduce the incidence of rebound pain and improve patient satisfaction. Studies have indicated that the administration of dexamethasone as a single intravenous dose or as an adjunct to local anesthetics can effectively reduce the occurrence of rebound pain and enhance patient satisfaction. Alternatively, using continuous nerve blocks or combining multiple nerve blocks has a lower probability of causing rebound pain compared to single nerve blocks [79–82]. Novel formulations of local anesthetics may also be considered, such as sustained release (liposomal) bupivacaine [83].

### Nerve damage after peripheral nerve blocks

Some researchers have reported an incidence rate of approximately 1 in 3000 for nerve damage following peripheral nerve blocks. While the incidence is relatively low, it can still lead to serious adverse outcomes [84]. The mechanism of nerve damage following peripheral nerve blocks is not yet fully understood; it seems to result from a complex interplay among patients’ factors, environmental factors (regional anesthesia tools and methods), and causative agents (mechanical and chemical) [85]. Most nerve injuries are considered to be secondary to intraneural injection, particularly at high pressure, and are felt to result in greater risk of nerve damage [86]. And almost all local anesthetics have neurotoxic and cytotoxic effects, when injected into the nerve or at high concentrations, which may lead to nerve cell damage [87]. Currently, no technique can definitively reduce the incidence of nerve damage. However, anesthesiologists can take a series of measures to minimize the likelihood of nerve damage during clinical procedures. These measures include using ultrasound guidance whenever possible, avoiding intraneural injections, using short-beveled needles (e.g., with bevel angles > 45°), avoiding high-concentration local anesthetics, and considering the use of pressure-monitoring syringes to control injection pressure below 15 psi (100 kPa) [88]. Therefore, prior to performing peripheral nerve blocks, comprehensive communication with the patient, including discussion of relevant risks and complications, is essential. Additionally, follow-up care should be provided to patients to promptly identify potential nerve damage and initiate early intervention.

### Fall risk after nerve blocks

While nerve blocks provide pain relief, but some lower extremity nerve blocks may result in muscle weakness which can secondarily increase the risk of postoperative falls. Prospective data from Canada showed that femoral nerve blocks with 0.5% ropivacaine can lead to quadriceps muscle weakness, which can result in slowed movement and an increased risk of falls for patients [89]. In trauma centers, where peripheral nerve blocks are administered to trauma patients, effective monitoring may be lacking, potentially resulting in limb or other site injuries. A double-blind randomized controlled study showed that use femoral nerve block using 0.1% ropivacaine combined with 2 µg/kg dexmedetomidine achieved good analgesia without affecting the patients’ lower limb muscle power [90]. Therefore, it is maybe a better choice to use low concentrations of local anesthetics for pain management.

## Conclusion and future prospects

Trauma has become a significant public health concern, with over 500 million people suffering injuries worldwide every year, of which more than 25% experience orthopedic injuries requiring surgical treatment [91]. To ensure timely and effective treatment for trauma patients, reduce mortality and disability rates, the National Health Commission of China called for the establishment of trauma emergency centers at all levels to centralize in-hospital treatment for trauma patients in 2018 [92]. Besides life-threatening conditions associated with trauma, acute pain management is a significant challenge. Anesthesia teams, as an essential part of multidisciplinary teams in trauma centers, are responsible not only for providing anesthesia and life support but also for acute pain management. Ultrasound-guided nerve block is an important part of perioperative pain management, which has the advantages of reducing patients’ pain, decreasing the use of opioids, reducing the occurrence of postoperative delirium, and shortening the length of hospitalization and reducing hospitalization costs. Ultrasound-guided nerve blocks have been widely used in intraoperative anesthesia or postoperative pain management, but have not been widely used in the early management of acute pain in trauma patients.

This article summarizes ultrasound-guided nerve block techniques that can be performed by anesthesiologists in the supine position in trauma centers for trauma patients, providing a new approach to acute pain management. However, ultrasound-guided nerve blocks performed in trauma centers carry the risk of adverse effects and complications, including but not limited to incomplete block, masking of patient condition, local anesthetic toxicity, “rebound pain” after nerve block, and re-injury after nerve block. This places high demands on the anesthesiologist’s ultrasound-guided nerve block technique, and also requires close monitoring of the patient’s condition by the surgical and nursing teams involved in the treatment.

The safe and effective implementation of ultrasound-guided supine nerve blocks for limb pain management in emergency orthopedic trauma patients warrants further exploration and research.

## Data Availability

No datasets were generated or analysed during the current study.
